# High-performance flat-type InGaN-based light-emitting diodes with local breakdown conductive channel

**DOI:** 10.1038/s41598-019-49727-4

**Published:** 2019-09-20

**Authors:** Seung-Hye Baek, Hyun-Jin Lee, Sung-Nam Lee

**Affiliations:** Department of Nano-Optical Engineering, Korea Polytechnic University, Siheung, 15073 Republic of Korea

**Keywords:** Electrical and electronic engineering, Inorganic LEDs

## Abstract

Flat-type InGaN-based light-emitting diodes (LEDs) without an n-type contact electrode were developed by using a local breakdown conductive channel (LBCC), and the effect of the In content of the InGaN quantum wells (QWs) on the local breakdown phenomenon was investigated. Electroluminescence and X-ray analyses demonstrated that the homogeneity and crystallinity of the InGaN QWs deteriorated as the In content of the InGaN QWs increased, thereby increasing the reverse leakage current and decreasing the breakdown voltage. After reverse breakdown with a reverse current of several mA, an LBCC was formed on the GaN-based LEDs. The surface size and anisotropic shape of the LBCC increased as the indium content of the InGaN QWs in the LEDs increased. Moreover, a flat-type InGaN LED without an n-type electrode was developed by using the LBCC. Notably, the resistance of the LBCC decreased with increasing indium content in the InGaN QWs, leading to lower resistance and higher light emission of the flat-type InGaN-based LEDs without an n-type contact electrode.

## Introduction

III-nitride semiconductors are promising materials that cover the ultraviolet and visible regions of the spectrum^1–3^. Recently, nitride-based semiconductors have been extensively studied for the development of high-performance light-emitting diode (LED) displays, solid-state lighting sources, telecommunications, and high mobility devices^4–7^. Many researchers have focused on improving the optical and electrical performance of these semiconductors to achieve higher optical output power and reliability^5–7^. To achieve high-performance LEDs, it is necessary to achieve high reliability factors related to the heat dissipation, junction temperature, surface recombination, and reverse leakage current^8–12^. The reverse leakage and breakdown characteristics are key parameters for obtaining highly reliable LEDs. In nitride-based semiconductors where GaN and its hetero-structure are grown on a foreign substrate (such as silicon carbide or sapphire) with a large lattice mismatch, the leakage current and breakdown become important issues due to crystal defects^13–16^.

To date, there is a strong focus on improving the reverse leakage and breakdown to reduce electrostatic discharge damage and to improve the long-term reliability of LEDs^17–19^. In general, the conventional breakdown phenomenon refers to full breakdown, where the LED is completely destroyed, leading to the loss of typical diode *I−V* characteristics. In our previous study, we developed a local breakdown technique that partially destroys the LEDs, rather than inducing general full breakdown^20^. The local breakdown could be induced directly by surface V-shape defects^20^, which may be related to the indium content of the InGaN QWs. It is well known that surface V-shape defects can be generated from growth of the InGaN active region because of the low mobility of surface adatoms around threading dislocations at relatively low InGaN growth temperatures^21–26^. Therefore, we focused on the effect of the indium composition of InGaN quantum wells (QWs) on the reverse characteristics (leakage current and breakdown voltage) and local breakdown phenomenon (such as series resistance and anisotropic surface morphology) of GaN-based LEDs. Based on these results, we herein evaluate the optical and electrical properties of new paradigm flat-type LEDs without an n-type contact electrode by using the local breakdown conductive channel (LBCC).

## Characterization of Optical Properties and Crystallinity of InGaN-Based LEDs with Variation of the Indium Content of the InGaN QWs

**Figure 1:**
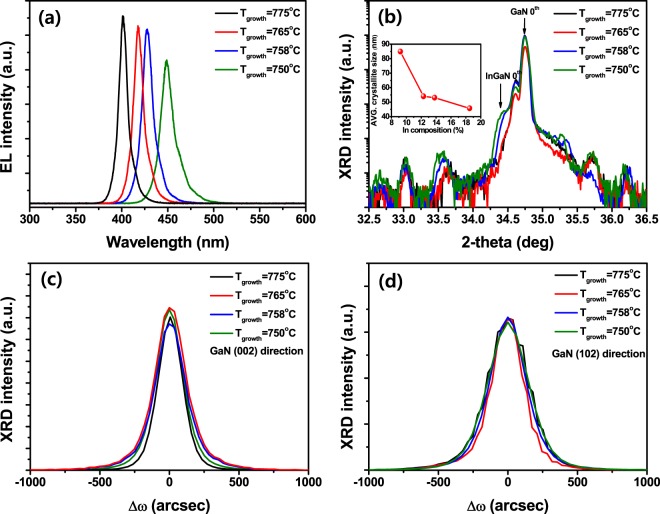
Optical and structural characteristics of InGaN-based LEDs with different InGaN QWs. EL spectra (**a**), X-ray (002) ω/2θ scans (**b**), ω-rocking (002) (**c**), and (102) (**d**) curves of InGaN-based LEDs employing InGaN QWs grown at different temperatures. Inset shows the average crystallite size calculated from Scherrer equation by using the InGaN 0^th^ peaks of HR-XRD ω/2θ scan for different indium concentrations.

Figure 1(a) shows the electroluminescence (EL) spectra of InGaN-based LEDs with InGaN QWs grown at different temperatures. As the growth temperature of the InGaN QWs increased from 750 to 775 °C, the emission wavelength of the InGaN-based LEDs was shortened from 450 to 400 nm. Because the growth temperature of InN is lower than that of GaN due to the volatility of In^27^, the In content of the InGaN active layer is almost inversely proportional to the growth temperature. Moreover, it is known that the indium incorporation coefficient (*k*_*In*_) increases with decreasing growth temperature and increasing growth rate at a constant growth temperature^28,29^. The higher indium incorporation efficiency achieved at lower temperatures has been attributed to reduced evaporation of indium atoms from the surface of the InGaN layer because the vapor pressure of indium is higher than that of gallium^29^. Therefore, the emission wavelength of the LEDs increased in proportion to the decrease in the growth temperature, as shown in Fig. 1(a). Moreover, as the growth temperature of the InGaN QWs decreased, the intensity of the emission decreased and the full width at half maximum (FWHM) of the emission spectrum became broader.

The indium compositions of the InGaN QWs grown at different temperatures were calculated from the high-resolution X-ray diffraction (HR-XRD) ω/2θ scan, as shown in Fig. 1(b), demonstrating that the indium content of the InGaN QWs is inversely proportional to the growth temperature. Furthermore, the FWHM of the InGaN 0th peak in the ω/2θ scan increased with higher indium content in the InGaN QWs, as shown in the inset of Fig. 1(b). As the indium content of the InGaN ternary compound increases, crystal imperfections may be formed by phase separation of indium due to the immiscibility gap of the InN-GaN ternary system^30^; these imperfections can operate as a non-radiative recombination centers^31,32^. Thus, it was judged that decreasing the growth temperature of the InGaN QWs led to less crystal inhomogeneity of the InGaN QWs, thereby decreasing the EL intensity and broadening the emission spectrum. X-ray rocking curves (XRCs) were acquired with two different X-ray incident beam directions (GaN (002) and GaN (102)), as shown in Fig. 1(c,d), respectively. In general, it is known that the FWHM of the XRC peaks of symmetric GaN (002) is mainly influenced by screw or mixed dislocations, but is insensitive to pure edge dislocations, whereas the FWHM of asymmetric GaN (102) is strongly affected by both the edge and mixed dislocations^33,34^. However, the FWHMs of the XRC peaks along the GaN (002) and GaN (102) directions were not directly affected by the growth temperature of the InGaN QWs. The average crystallite size of the InGaN active layer was evaluated by applying Scherrer equation^35^. The FWHM of the InGaN 0^th^ peak increased with the indium content, indicating that the average crystallite size of the InGaN layer increased with higher indium incorporation shown in inset of Fig. 1(b). This observation implies that the crystal defects in the InGaN QWs increase with the indium concentration due to the greater fluctuation of the indium content or lattice mismatch-induced strain. Thus, it is deduced that increasing the indium concentration in the InGaN QWs did not significantly increase the threading dislocations in the GaN film, but could affect crystal imperfections in the InGaN active layer.

## Reverse Leakage and Breakdown Characteristics of InGaN-Based LEDs Employing InGaN QWs with Different Indium Compositions

**Figure 2:**
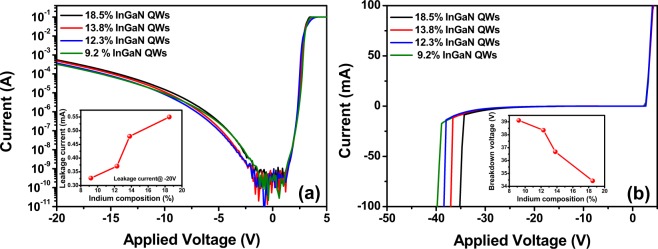
I-V characteristics of InGaN-based LEDs with different indium contents of InGaN QWs. Logarithmic (**a**) and linear (**b**) scale *I-V* curves of InGaN-based n-p LEDs with InGaN QWs having different indium compositions. Insets of (**a**,**b**) show the reverse leakage current at −20 V and the breakdown voltage as a function of the indium composition in the InGaN QW region, respectively.

Figure 2 shows the reverse leakage and breakdown characteristics of the InGaN-based n-p LEDs with variation of the indium content of the InGaN QWs. The reverse leakage current increased as the indium content of the InGaN QWs increased, as shown in Fig. 2(a). In general, it is known that the reverse leakage current is significantly affected by crystal defects^36^. The X-ray results showed exacerbation of the crystal inhomogeneity of the InGaN QWs with increasing indium concentration due to the fluctuation of the indium composition. Therefore, it is proposed that the increased reverse leakage current results from crystal imperfections around the InGaN QWs due to the indium localized inhomogeneities. Figure 2(b) shows that the reverse breakdown voltage decreased with increasing indium concentration in the InGaN QWs. These results are consistent with the reverse leakage current trends. It is known that reverse breakdown can be significantly affected by crystal defects such as threading dislocations, surface V-shaped defects, etc^35,37–39^. However, the additional formation of two-dimensional crystal defects did not increase significantly after growth of the InGaN QWs with different indium compositions, as mentioned in relation to the XRC analysis. Therefore, we surmise that this breakdown is significantly affected by the increased crystal inhomogeneity generated by the high electrical field in the InGaN/GaN QWs with high indium contents. Furthermore, it is reported that local breakdown can occur at the surface V-shaped defects^20^. Such surface V-shaped defects can be easily formed with increasing indium content in the InGaN QWs due to short migration of the surface adatoms at the relatively low growth temperature of InGaN^40^. Therefore, it is deduced that the local breakdown voltage decreases as the indium content of the InGaN QWs increases.

## Surface Analysis of Local Breakdown Conductive Channel in InGaN-Based LEDs

**Figure 3:**
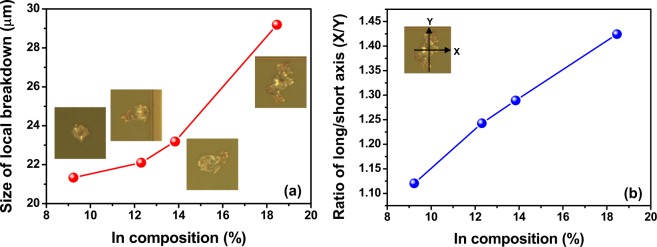
Surface analyses of local breakdown conductive channel on the InGaN-based LEDs with different InGaN QWs. Average surface size (**a**) and size ratio of long/short axis (**b**) of local breakdown in InGaN-based LEDs with variation of the indium content of the InGaN QWs. Insets show optical microscope images of local breakdown conductive channel in the InGaN-based LEDs.

Figure 3(a) shows the optical microscope images of the local breakdown regions after reverse local breakdown of the InGaN-based LEDs employing the InGaN QWs with different indium compositions. The images show an increase in the size of the local breakdown region in the longitudinal direction as the indium concentration of the InGaN QWs increased. Moreover, the long-axis to short-axis ratio in the local breakdown region increased as the indium concentration of the InGaN QWs increased. Micro-Raman analysis of the surface V-shape defects, which represents the Stokes shift for the GaN peak shown in Fig. S1, indicated that the surface V-shape region exhibits a tensile stress state that can easily lead to reverse breakdown of the GaN film. Therefore, it is proposed that local breakdown may occur in the valley region of the V-shaped defects for the InGaN QWs with a high indium content, leading to an increase in the size of the local breakdown region as the indium content of the InGaN QWs increases. Regardless of the indium composition of the InGaN QWs, the surface size of the V-shape defect was very small (<3.0 μm) for the GaN-based LEDs. However, as the indium content of the InGaN QWs increased, the size of the local breakdown region increased from 21.3 to 29.2 μm. The depth of the local breakdown was less than 1.0 μm, which penetrates a part of the n-GaN layer and all of the InGaN QW and p-GaN layers shown in Fig. S2(a). This observation implies that the indium composition of the InGaN QWs may affect the anisotropic surface size rather than the depth of the local breakdown. Further, the long-axis to short-axis ratio of the local breakdown surface increased with increasing indium content in the InGaN QWs, as shown in Fig. 3(b). This demonstrates that as the indium content of the InGaN QWs increased, the surface morphology of the local breakdown changed from isotropic to anisotropic. We speculate that a high indium content in the InGaN QW region can exacerbate irregular indium phase separation, thus increasing the anisotropic local breakdown surface due to the anisotropic tensile stress^41^.

## Optical and Electrical Analysis of the N-P InGaN-Based LEDs with LBCC

**Figure 4:**
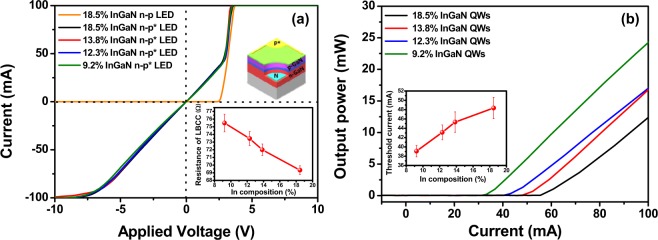
*L-I-V* characteristics of InGaN-based n-p and n-p* LEDs with different InGaN QWs. *I-V* (**a**) and *I-L* (**b**) curves of local breakdown n-p* InGaN-based LED with variation of the indium content of the InGaN QWs, where * is the local breakdown region. Insets of (**a**,**b**) show resistance and the threshold current of local breakdown InGaN-based LEDs as a function of indium content of InGaN QWs, respectively.

Figure 4(a,b) show the current (*I*)–voltage (*V*) and light output power (*L*)–current (*I*) profiles for the InGaN-based LEDs with variation of the indium content of the InGaN QWs after formation of the local breakdown regions, respectively. The local breakdown LED exhibits almost linear (conductive-like) properties below +3.4 V and the normal *I–V* characteristics of an n–p LED above 3.4 V. This *I−V* behavior of the local breakdown LED can be attributed to saturation of the carriers through the local breakdown channel within a limited channel around 3.4 V and overflow to other non-breakdown regions above 3.4 V^20^. The resistance of the LBCC of the InGaN-based LEDs was calculated from the *I−V* curves in the region below 3.4 V, as shown in the inset of Fig. 4(a). The resistance of the LBCC decreased slightly with increasing indium content in the InGaN QWs. It is thus postulated that the resistance of the LBCC is related to the size of the local breakdown region based on comparison with the size of the local breakdown shown in Fig. 3. Thus, the resistance of the LBCC decreases as its surface area increases.

EDX line profile analysis was performed on the In atoms near the edge of the LBCC region, indicating a high concentration of In near the boundary shown in Fig. S2(b,c). From these results, we believe that the conductance of the LBCC originates primarily from the indium atoms in InGaN and the ITO films in the LEDs. When local breakdown occurs under high reverse bias, the ITO, InGaN, and GaN layers decompose into the elemental components such as Ga, In, N, Sn, and O. After decomposition, some elemental atoms can be re-deposited or diffuse at the edge region of the LBCC, thus forming a conductive metal layer at the LBCC. Among the few conductive elemental atoms, we believe that indium may be a major source of the LBCC, connecting the n-GaN layer as a parallel resistance, based on the existence of indium atoms at the edge of the LBCC and the indium composition-dependence of the parallel resistance shown in the inset of Fig. 4(a). At this point, further study of the conductive mechanism of the LBCC is needed. The emission threshold current of the InGaN-based LEDs was observed by forming a local breakdown region. Because the LBCC can operate as a parallel resistance that bypasses the n-p junction, there is no emission until the LBCC is saturated at 3.4 V, as shown in Fig. 4(b). Based on these results, we suggest that the emission threshold current of the InGaN-based LEDs with the LBCC can be reduced by decreasing the indium content of the InGaN QWs.

## High-Performance Flat-Type Local Breakdown InGaN-Based LEDs without N-Type Contact Electrode

**Figure 5:**
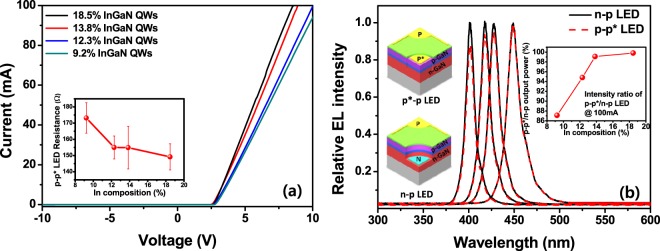
Electroluminescence analyses of InGaN-based n-p and flat-type p-p* LEDs with different InGaN QWs. *I−V* (**a**) curves of n-type electrode-free p-p* LEDs with different indium compositions and relative light output power ratio at 100 mA, (**b**) relative EL spectra of n-p and p-p* LEDs with variation of the indium content of the InGaN QWs. Inset of (**a**) shows indium composition-dependent resistance, and insets of (**b**) show light output power ratio of p-p* to n-p LEDs as a function of indium content of InGaN QWs.

InGaN-based LEDs were fabricated without an n-type contact electrode by utilizing the LBCC that can function as an n-type contact electrode, as shown in the inset of Fig. 5(b). Figure 5(a) shows the *I−V* curves of the n-type contact-free InGaN-based LEDs with different indium compositions in the InGaN QW region. Despite the n-type contact electrode-free LED structure, the turn-on voltage was clearly 2.65 V for all samples. However, the series resistance of these LEDs declined in inverse proportion to the indium content of the InGaN QWs, which is consistent with the resistance data for the LBCC in the InGaN-based LEDs with different indium compositions shown in the inset of Fig. 4(a). Figure 5(b) shows the relative EL spectra of the n-p and p-p* LEDs with different indium compositions in the InGaN QWs. In order to exclude the effect of the indium content of the InGaN QWs on the light output power of the LEDs with the LBCC, the light output power ratio of a conventional n-p LED was compared with that of the local breakdown p*-p LED as a function of the indium content (see inset of Fig. 5(b)), where p* is the p-contact layer, with the LBCC acting as an n-type contact layer. The series resistance of the p-p* LEDs increased with decreasing indium content in the InGaN QWs (Fig. 5(a)), resulting in a resistance heating effect and consequent decrease in the EL intensity of the p-p* LEDs. As a result, the light output power ratio increased when the indium content of the InGaN QWs was higher. Therefore, we suggest that relatively high photoemission can be achieved with the p*-p flat-type LEDs having a high indium content in the InGaN QWs, where the performance is comparable to that of conventional n-p LEDs due to reduction of the series resistance originating from the lower resistance of the LBCC. However, the series resistance of the p-p* LEDs with the LBCC is still higher than that of conventional n-p LEDs, which may lower the emission efficiency, reliability, etc. Thus, further studies on decreasing the series resistance of the LBCC in the p-p* LEDs are required.

## Conclusions

We investigated the effect of the indium content of the InGaN active region on local breakdown phenomenon and flat-type InGaN-based LEDs without an n-type contact electrode. The reverse leakage current was higher for the InGaN-based LEDs with a higher In content in the InGaN QWs, whereas the breakdown voltage was lower. The surface size and anisotropic shape of the local breakdown region increased when the indium content of the InGaN QWs was higher. The surface morphology of this local breakdown is affected by tensile stresses generated from indium segregation in the high In composition InGaN QWs. Moreover, we evaluated the electrical and optical properties of InGaN-based LEDs without an n-type contact electrode, presenting a new paradigm of flat-type p*-p LEDs using LBCC. *I−V* analysis of the conventional n-p LED with LBCC showed that the resistance of the LBCC decreases when the indium content of the InGaN QWs is increased. Thus, as the indium content of the InGaN QWs increases, the low series resistance results in a flat-type p-p* LED with higher light output power.

## Methods

InGaN-based LED epitaxial layers were grown on (0001) c-plane sapphire using metal-organic chemical vapor deposition. Trimethylgallium and ammonia (NH_3_) were used as Ga and N sources, respectively. The LED epitaxial structure is composed of 3.0 μm thick undoped GaN, 2.0 μm thick n-type GaN, 30 nm thick InGaN, five period InGaN/GaN QWs, a 20 nm thick Mg-doped AlGaN electron blocking layer, and 150 nm thick p-GaN layer. The growth temperature of InGaN QWs was controlled from 750 °C to 775 °C to investigate the effect of the indium content in the InGaN/GaN QWs on the reverse characteristics of the LEDs. After growing these epi-structure wafers, LED chips with a size of 450 μm × 450 μm were fabricated by a conventional mesa LED process with lateral n-p and p-p electrode structures, as shown in the insets of Fig. 5(b). To form the local breakdown region in the p-layer, the anode and the cathode current were applied to the p_1_ and p_2_ layers, respectively. When a low injection current (~0.3 mA) was applied, a very high operation voltage of up to –50 V was observed, which was similar to the behavior of conventional n–p LEDs under reverse bias. However, when the applied current exceeded 1.0 mA, the operation voltage of the p–p LED structure decreased suddenly from 53.5 to 2.75 V. This voltage-drop continued as the applied current was increased and persisted even after the injection current was reduced to below 1.0 mA, indicating formation of the local breakdown (p*) region under these conditions^20^.

We evaluated the crystallographic properties of four LEDs with InGaN/GaN active layers with different contents by using a Rigaku DMAX 2200 HR-XRD instrument. The indium composition of the InGaN QW, as the active layer, was calculated by using Vegard’s law^42,43^. EL measurements were performed to observe the emission properties of four InGaN-based LEDs employing InGaN QWs grown at different temperatures. To evaluate the electro-optical characteristics, light output power-current-voltage (*L−I−V*) measurements were performed by using an HP4155 parameter analyzer. The effect of the In content of the InGaN QWs on the reverse leakage current and local breakdown phenomena in the LEDs was monitored by increasing the reverse bias voltage.

## Supplementary information

Supplementary information
