# Supplementary material for: High-performance flat-type InGaN-based light-emitting diodes with local breakdown conductive channel
Source: Sci Rep. 2019 Sep 20;9:13654. doi: 10.1038/s41598-019-49727-4 (PMC6754497; doi:10.1038/s41598-019-49727-4)
Supplement: Supplementary file 1 — Supplementary information [file 41598_2019_49727_MOESM1_ESM.docx]

**Supplementary Information**

**High-performance flat-type InGaN-based light-emitting diodes with local breakdown conductive channel**

Seung-Hye Baek, Hyun-Jin Lee & Sung-Nam Lee*

Department of Nano-Semiconductor Engineering, Korea Polytechnic University, Siheung 15073, Republic of Korea

**S1. Mirco-Raman analysis of surface V-shape defect region of InGaN-based LEDs**

**S2. HR-TEM analysis of LBCC region in the n–p* GaN-based LEDs**

**S1. Mirco-Raman analysis of surface V-shape defect region of InGaN-based LEDs**

**
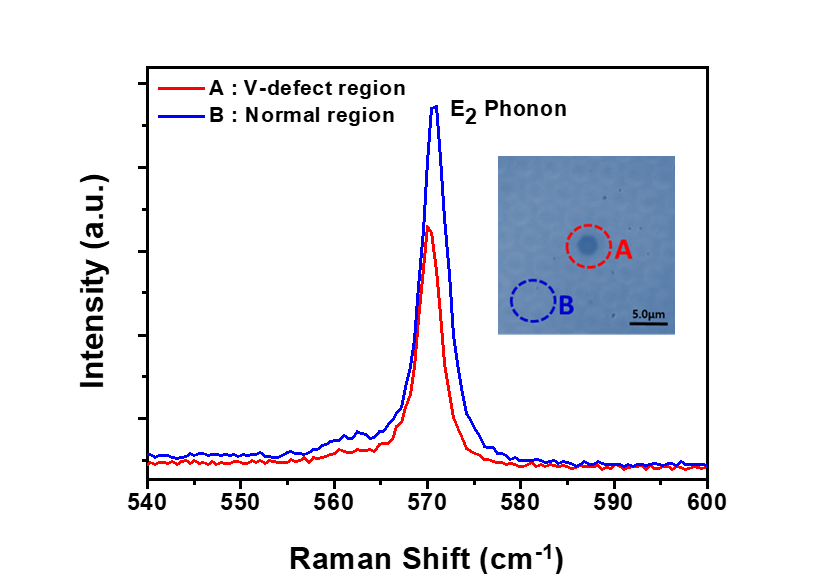
**

**Figure S1** Micro-Raman shifts of surface V-defect region (Position A) and normal region (Position B) on the GaN-based LED. Inset shows the optical microscope image of both regions.

We measured the stress status of surface V-defect region on GaN-based LEDs by using micro-Raman spectroscope. GaN-related E2 phonon peaks of both regions were clearly observed by shown in Fig. S2. It shows that the Raman shift of the normal region in GaN-based LED is 570.7cm^-1^, but the Raman shift of V-shape defect region is 570.2cm^-1^. The surface V-defect region exhibits a relative higher tensile stress than other normal surface region. Therefore, we speculate that the local breakdown conductive channel can be predominately formed at the surface V-defect region.

**S2. HR-TEM analysis of LBCC region in the n–p* GaN-based LEDs**


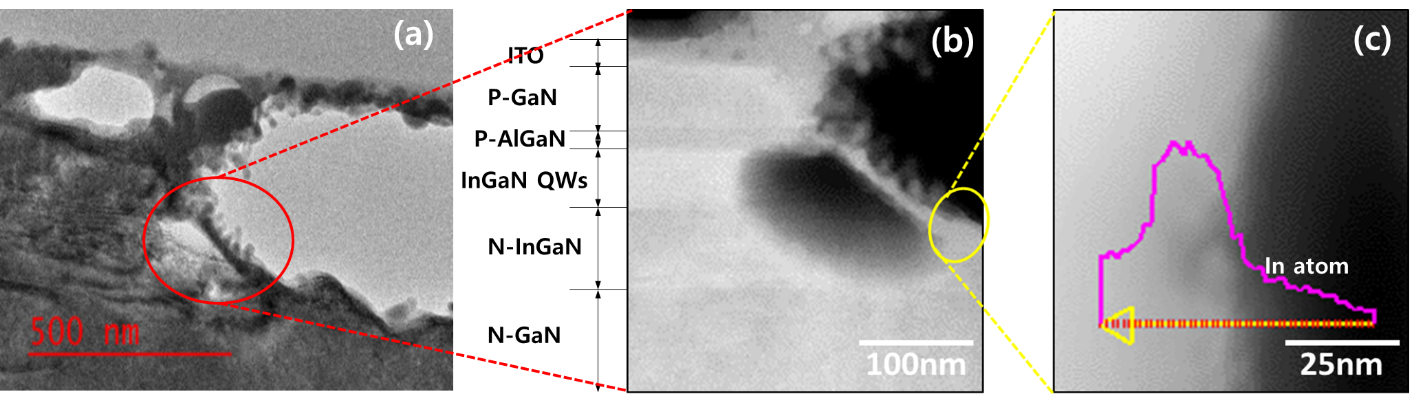


**Figure S2** (a) The cross-sectional HR-TEM image of GaN-based LEDs with LBCC region, (b) the STEM image of LBCC region with n-GaN, QWs and p-GaN epilayers, and (c) the EDX line profile of indium atoms in the LBCC.

Through the HR-TEM analysis, we observed a cross-section TEM image and EDX line profile of the local breakdown region. In particular, a line profile was performed on the In atom near the edge of the LBCC region, indicating that the high In distribution was observed near the boundary. Therefore, we believe that main conductive origin of LBCC can be indium atom contained in InGaN and ITO films in LEDs. When local breakdown occurs under the high reverse bias, ITO, InGaN and GaN layers can be decomposed into elemental atoms such as Ga, In, N, Sn and O. After decomposing process, some elemental atoms can be re-deposited or diffused at the edge region of LBCC, which is the formation of conductive metal at the LBCC. Among a few conductive elemental atoms, we believe that indium atom can be a major source of the LBCC connecting n-GaN layer as a parallel resistance because of the existence of indium atom at the edge of LBCC and the indium composition dependence of parallel resistance shown in inset of Fig. 4 (a). In this point, we will need to further study the conductive mechanism of LBCC in detail.
